# The Moderating Effect of Social Support between Loneliness and Depression: Differences between the Young-Old and the Old-Old

**DOI:** 10.3390/ijerph19042322

**Published:** 2022-02-17

**Authors:** Hyegyeong Son, Heeran J. Cho, Sunghwan Cho, Juhyun Ryu, Sunghee Kim

**Affiliations:** 1 College of Nursing, Kosin University, Busan 49104, Korea; hkprin@kosin.ac.kr; 2 Department of Health Administration, Yonsei University, Seoul 03021, Korea; 3 School of Social Work, Virginia Commonwealth University, Richmond, VA 23284, USA; chos11@vcu.edu; 4 Graduate School of Social Welfare, Yonsei University, Seoul 03722, Korea; jun.r.openbox@gmail.com (J.R.); ohmyenergy@gmail.com (S.K.)

**Keywords:** social support, loneliness, depression, older adults, CES-D

## Abstract

This study aimed to investigate the moderation of social support in the association between loneliness and depression in different age groups of older adults. The sample consisted of 1532 community-dwelling adults aged 65 years or older, based on the data from the National Social Life, Health, and Aging Project (NSHAP), Wave 3 (2015–2016). Eleven items of the Center for Epidemiologic Studies Depression Scale (CES-D) were used to measure depressive symptoms. Similarly, a four-item scale was used to measure social support (each from spouse and family), and a three-item scale for loneliness. The results were as follows. Loneliness was associated with depression in both the young-old and the old-old groups. Spousal support and family support were associated with reduced depression in the young-old group, whereas only spousal support was associated with relieving depression in the old-old group. Social support had a significant moderating effect on the relationship between loneliness and depression in the old-old group, whereas it had no significant effect in the young-old group. From these results, it can be concluded that spousal support plays a significant role in seniors’ mental health. The role of caregivers for a person’s well-being grows later in life, so practitioners could help couples communicate with this consideration. In addition, regular contacts with family members and spousal support are recommended to prevent older old adults’ depression.

## 1. Introduction

Depression is one of the most common factors that diminishes the quality of life among older adults [1], both because of its impact and frequent comorbidity in late life. Steinman et al. [2] estimate that seven million American adults aged 65 or older are affected by depression. The prevalence of depression among community-dwelling adults above 65 is estimated to be between 5% and 10% [1]. Comorbidity of depressive disorders in older patients have been documented with a number of chronic illnesses and functional declines, such as Alzheimer’s disease [1]. It is also one of the major risk factors of disability, premature mortality [3], and even suicide, but depression is frequently underdiagnosed in older adults [4,5]. Furthermore, research has indicated depression’s association with reduced physical functioning, greater pain, and diminished mental health [2,6].

Loneliness is defined as the subjective perception of being socially isolated, resulting from a discrepancy between one’s social needs and relationships [7,8]. It is one of the main factors leading to depression [9,10], and is a predictor of many health and behavioral problems, including alcoholism, higher blood pressure, and sleep issues [10,11,12,13]. Other researchers also found strong associations between loneliness and adverse mental health, including anxiety and depression [14,15]. Loneliness is especially considered to have a close relationship with, or to be a risk factor of, depression, because events in later life, such as retirement and deaths of a spouse or friends, may contribute to loneliness, which may also occur with depression [10].

It was recently discovered that cultural factors influence personal well-being. Cultural factors, including individualism and collectivism, can help to enrich the understanding of related factors of loneliness and depression as they can examine relations of cultures of countries, society, and people. The evidence for the effects of individualism and collectivism on quality of life is somewhat mixed. There is a report that individualism positively affects the national average subjective well-being while pursuing personal happiness and creating an open society with tolerance and trust for oneself [16]. A study showed that propensity reduces well-being [17]. Although these findings have implications in that individualism and collectivism provide information on life stages, cultural factors, and well-being, it is still essential to understand life stage (age), psychological, and economic factors together to understand well-being at the individual level. In particular, economic factors are known to be strongly associated with or to amplify loneliness and depression.

The association between loneliness and depression among older adults has been thoroughly documented. Loneliness is likely to increase with more age-related losses, resulting from lack of connection with family and community, plus reduced physical mobility [10]. Loneliness and depression are significantly and positively correlated in older adults [10], in addition to other significant correlates, including social support, religiosity, and exercise [11], and demographic factors such as age, gender, ethnicity, and education [12]. Loneliness was found to be correlated to depression cross-sectionally, and to have a causal influence on depression over time [18]. The association between loneliness and depression is even more critical to later-life health because they can work synergistically to lower the well-being of older adults [19].

The fact that loneliness co-occurs with depression among older adults is closely related to their reduced social participation. Diminished ability in later life to engage in and maintain social networks, or loss of desired intimacy, may result in chronic late-life depression, which may damage relationships and result in further isolation [20]. In a study, homebound older adults scored higher depressive symptoms than their counterparts in senior centers, due to the services and meals provided in senior centers and more frequent interaction with the peers and staff members [21]. Their limited mobility may have caused less help-seeking activities, which were only limited to consulting their physicians and social workers, not professionals. According to Houtjes et al. [22], decreasing social network size and increased feelings of loneliness may lead to further depression, putting seniors in a vicious cycle that undermines the sense of belonging and leads to social isolation; this is more evident among vulnerable older adults. In particular, loneliness significantly contributed to the presence of depressive symptoms among older residents of subsidized housing, who are disproportionately people of color [3]; subpar living conditions of public housing may contribute to a higher rate of loneliness and depression, along with various vulnerabilities associated with living in public housing.

Risk factors, including certain demographics such as gender and age [19], socioeconomic factors such as limited economic resources, and psychological vulnerabilities such as low social support and high perceived stress [18], are closely associated with either loneliness or depression, or amplify the correlation of the two. The association between loneliness and depression may be more pronounced in low-education and low-income groups, because older adults who experience financial pressures and worries are unable to seek socialization and employ appropriate coping resources. However, Choi and McDougall [21] reported the importance of social support as a major coping resource because coping with their stressors heavily depends on the availability of appropriate resources. They found that social support mitigated the harmful effects of individual’s financial situation and physical impairment on depression for homebound older adults. The fact that situations such as dependence on others or mobility limitations are no longer risk factors when appropriate coping and resources are accounted for shows that stressful life events, their appraisal, and coping are inextricably connected.

Social support is known to be one of the most crucial protective factors against depression for older adults [20]. Social networks with adequate size, quality, and interaction frequency give their members the feeling of being valued and loved [23]. Support from family and friends moderated psychological stress, promoted health, and prevented loneliness and depression later in life [21,24]. Supports are meaningful when provided to the elderly, and are reciprocal because reciprocity of support gives seniors a sense of security, relief, and eventually higher life satisfaction [25]. The source of social support could come from different people, and intimate bonds with the spouse and/or family are considered to be particularly protective against depression. More frequent partner loss may elevate the prevalence of depression in older women, even when men who experienced partner loss were more vulnerable to depression [26]. However, support from different sources may result in different depression outcomes, especially when older adults are not comfortable with support from neighbors or when their circumstances hinder them from mutual support due to disability or financial issues [21]. Different sources of support may also be differently associated with loneliness, whether from social networks or marital relationships, the first of which is more closely associated with depression [19].

Cohen and Wills’ [27] Stress, Social Support, and Buffering Hypothesis suggests that social support works both as the main effect on health and buffers the effect of stress on health. Social network support helps improve the reinterpretation of stressful experiences and alleviates the stress reaction to the appraised stress of the event. It may be the mechanism through which social support intervenes between the pathway from loneliness to depression in older adults. Chen et al. [28] reported that social support promoted positive coping, alleviated negative coping, and moderated depression and loneliness. Lonely individuals are highly alert to threat information and more susceptible to depressive cognitive bias, so social support may protect older adults from falling into the vicious cycle of loneliness and depression augmenting each other [20]. Emotional, affectionate, and tangible support even predicts positive health behaviors via psychological mechanisms such as self-efficacy to counteract the negative effect of depression on health [29]. Older people with higher social support likely have more diverse external sources they can rely on for helpful information or models of positive health management, which is further evidence that social support prevents depression via better coping skills.

Demographic factors such as gender and age also factor into the association between loneliness, depression, and social support. The association between loneliness and depression was substantial for both older men and women. However, when perceived stress and social support were accounted for, the association only remained for older men [18]. This gender difference is closely linked to partner interaction; whereas older women’s perceived partner support was more associated with depression, older men’s association had to factor in their perceived independence and attachment to their wives [30]. Choi and Ha [31] found that lower partner support was associated with higher depression scores only among older women, and partner support was a more important correlate of depression among older men than women. Since life expectancy has increased, the need to define and categorize “elderly” in a new way has been argued. Age may alter the relationship among various variables associated with depression. Stressful life events may occur more frequently with age, such as financial difficulties, death of intimate people, and new disease or disability; all of these risk factors contribute to depression [32]. The old-old (aged 75 years and above) experience more geriatric frailty, dependence in everyday life, and risks of hospitalization than the young-old (aged 65–74 years), thus showing different predispositions in various aspects of life, including the levels of acculturation, acculturative stress, and depression. For the very old, defined 90 years old or above, social support was the strongest predictor for subjective well-being, and family support was the only factor affecting their morale [33].

**Figure 1:**
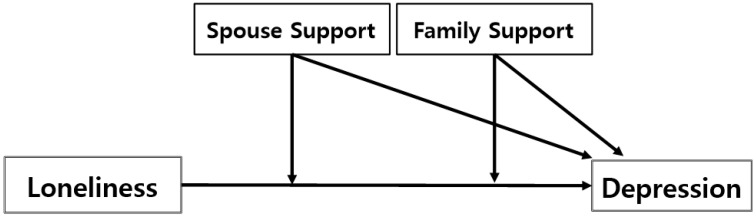
The Model of Moderation by Social Support.

This study aimed to examine the moderation of kinship social support (spouse/family) in the relationship between loneliness and depression (See Figure 1). Moreover, the moderation was investigated in two distinct groups: the young-old (age 65–74 years) and the old-old (age 75 years and above). This study attempts to answer the following questions: (1) Does the relationship between loneliness and depression differ by social support?; (2) What is the difference in the moderating effect of social support in the relationship of loneliness and depression between the young-old and the old-old?

By examining the moderation of social support between loneliness and depression among older adults, this study expands the limited information about the mental well-being of the elderly population. The results of this study may also provide valuable insights for social service providers and policymakers to develop mental health intervention programs targeting senior citizens.

## 2. Methods

### 2.1. Data

The source of the data used for this study was the National Social Life, Health, and Aging Project (NSHAP), Wave 3 (2015–2016). The NSHAP investigates health, social life, and well-being of older American adults, representing the whole population. Interviews were conducted in two languages, English and Spanish, conducted in-home and in-person, resulting in a total of 4377 community-dwelling respondents aged from 49 to 95. For the purpose of this study, samples aged below 65 were excluded, and only those aged 65 or older were used (*n* = 1532). The main variables used in this study were loneliness, depression, spouse/partner support, and family support. The cases with missing values and outliers from the main variables were further excluded, and the final sample was 903 young-old and 629 old-old respondents, which were included in the analysis.

### 2.2. Measures

#### 2.2.1. Dependent Variable: Depressive Symptoms

Depressive symptoms were measured by the modified 11-item Center for Epidemiologic Scale for Depression (CES-D) [34,35]. The scales range from 1 (rarely or none of the time) to 4 (most of the time), with a maximum possible score of 44. Given that a score of 16 or higher is commonly accepted as clinically significant depressive symptoms in the original 20-item scale, a score of 12 or higher was regarded as clinically significant depression in the modified scale. Among the 11 items, “I was happy” and “I enjoyed life” were reverse-coded. The scores were averaged, and higher scores indicate greater levels of depression. The Cronbach’s alpha for the study sample was 0.775.

#### 2.2.2. Independent Variable: Loneliness

The loneliness measure used in NSHAP is the widely used Revised University of California, Los Angeles (UCLA) Loneliness Scale (RULS) Short form [34,35]. The scale consists of three questions, and each item was rated on a 4-point scale ranging from 0 (never) to 3 (always). The total score was averaged, and a higher average score indicates greater loneliness. The Cronbach’s alpha for the RULS of the sample was 0.809.

#### 2.2.3. Moderator Variable: Social Support (Spouse/Partner, Family)

The social support scale in NSHAP uses Schuster et al.’s [36] social support scale to assess the level of social support from spousal/partner and family, and is designed to assess social support in intimate relationships. Participants were asked to rate their present level of social support on a 4-point scale (0 = never, 1 = almost never or rarely, 2 = sometimes, 3 = often). A total score of four questions was averaged. The question “How often does/spouse/partner/family make too many demands?” and “How often does spouse/partner/family criticize?” were reverse-coded in both spouse/partner support and family support measures. The Cronbach’s alpha of the social support scales in the sample was 0.624.

#### 2.2.4. Control Variable

The control variables in this study were gender (male = 0, female = 1), age (continuous variable), education attainment (less than high school = 1, high school equivalent = 2, vocational certificate/some degree/associates = 3, bachelor’s degree or more = 4), race (0 = white, 1 = other).

### 2.3. Data Analysis

Descriptive statistical analyses and frequency distribution analyses were performed, including means, standard deviations, ranges, and distributions, using the Statistical Package for Social Science, version 26. Independent sample t-tests were undertaken to compare main variables’ mean differences between the two subgroups of the young-old and the old-old. Multiple regression was implemented, and moderation analysis was performed with the SPSS macro PROCESS [37]. Multicollinearity among the predictors was further assessed with tolerance statistics and the variance inflation factors (VIF) [38] test to identify potential collinearity issues. The VIF value was less than 10, which indicates that there was no issue with multicollinearity among the independent variables.

## 3. Results

### 3.1. Sociodemographic Characteristics

Participants of this study consist of the young-old (*n* = 903) and the old-old (*n* = 629), as shown in Table 1. A share of 48.7% was female in the young-old group, and 40.2% were female in the old-old group. The mean age of the young-old group was 69.79 (SD = 2.58), and in the old-old group, 80.21 (SD = 4.17). Attainment of vocational certificate/some degree/associates was the most frequent education attainment level in the young-old (34%) and the old-old (31.2%). A share of 78.4% was white in the young-old group and 76.3% were white in the old-old group.

### 3.2. Differences of Main Variables between the Young-Old and the Old-Old

Independent t-tests were undertaken to identify mean differences of main variables, including loneliness, spouse support, family support, and depression. There were no statistically significant differences in these variables between the young-old and the old-old (See Table 2).

### 3.3. Multivariate Regression Analysis

**Figure 2:**
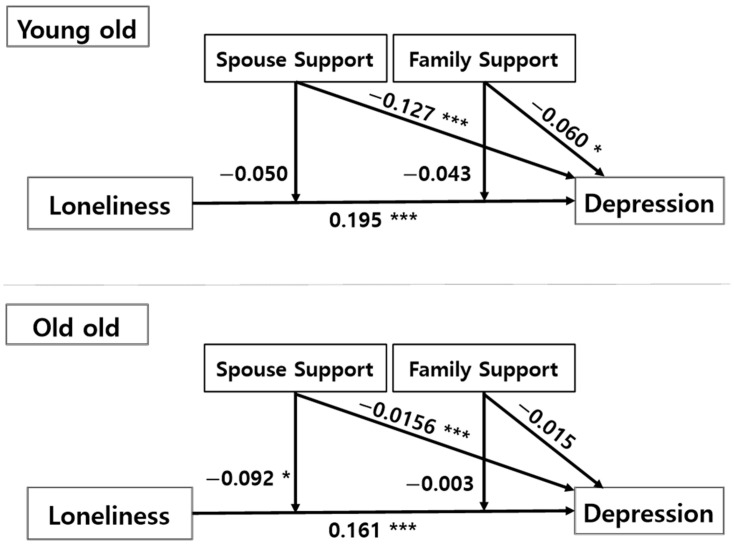
Model of Moderation by Social Support: Young-Old and Old-Old. Note: * *p* < 0.05, *** *p* < 0.001.

The moderating effect of spouse support and family support between loneliness and depression is shown in Table 3 and Figure 2. The regression analysis was statistically significant for the young-old group (F = 32.941, *p* < 0.001), and the model explained 24.9% of the variance in depression scores (R^2^ = 0.249). Demographic variables such as gender (B = 0.087, *p* < 0.01), education attainment (B = −0.055, *p* < 0.001), and race (B = −0.072, *p* < 0.05) significantly predicted depression. When the person was female, attained lower a level of education, and belonged to the non-white group, the person was likely to have a higher level of depression.

Both loneliness, the independent variable (B = 0.195, *p* < 0.001), and spouse support, the moderating variable (B = −0.060, *p* < 0.05), had a statistically significant effect on depression. The lonelier the person, and the less spousal and family support the person felt, the more depressed the person. Interaction terms were not significant, neither loneliness × spouse support or loneliness × family support, thereby indicating that spouse support and family support did not significantly moderate the effect of loneliness on depression.

For the old-old group, the model explained 18.7% (R^2^ = 0.187) of the variance in depression scores, and the model was statistically significant (F = 15.816, *p* < 0.001). Regarding the relationship between the main variables, demographic control variables such as gender (B = 0.089, *p* < 0.01) and age (B = 0.012, *p* < 0.01) had a statistically significant impact on depression. This means that when the person was female and older, the person was more likely to have higher depression. Both loneliness (B = 0.161, *p* < 0.01) and spouse support (B = −0.156, *p* < 0.001) significantly predicted depression. That is, the lonelier the person, the higher their depression score. However, family support was not a statistically significant predictor of depression. In addition, the loneliness × spouse support interaction term (B = −0.092, *p* < 0.05) had a significant effect on depression, but the loneliness × family support interaction term did not, indicating that different types of social supports had different implications for the old-old group’s depression.

## 4. Discussion

This study examined the moderation of social support on the association between loneliness and depression among older adults. Regardless of age group, it was found that less spouse/partner support can increase depression, but family support was only statistically significant in the young-old group. This study also addressed a significant gap in the literature by investigating the differences in such an association between the young-old and the old-old. To our knowledge, this is the first study to identify the effects based on the age of older adults.

In the young-old group, female or non-white senior citizens were more likely to experience depression. Moreover, older adults with less education attainment or more perceived loneliness experienced more depression. It was discovered that both spouse support and family support regulate the relationship between loneliness and depression in this group. The less spouse support or family support the young-old person perceived, the more depressive they were. On the other hand, in the old-old group, female older adults were also more vulnerable than male older adults, but there was no difference in the level of depression across different racial groups. Age and loneliness were significant predictors of depression in this group. Spouse support, but not family support, was also found to significantly predict depressive symptoms for this subgroup.

Some of the findings of this study confirm previous research, whereas others show inconsistencies. The finding that loneliness is associated with depression is consistent with previous studies on senior citizens aged 60 or older [39,40]. Furthermore, spousal support and family support had a negative impact on depression in the young-old group, whereas only spousal support relieved depression in the old-old group. This result is consistent with an earlier study that found that high-level spousal support in older adults was better protection against depression than that from their children and friends [41]. Nevertheless, the previous study failed to report spousal support’s effect in the old-old group. This study did not distinguish separate effects of individual predictors in different age groups, and studied the larger age group from 50 years old to 85 and older instead. In addition, there was a significant moderating effect of social support on the association between loneliness and depression among the old-old, whereas a similar effect was not found in the young-old group.

The results from this study offer suggestions to improve mental health services for older adults. Based on the evidence, the Centers for Disease Control and Prevention [42] recommends home- or clinic-based depression care management (DCM) programs for depression management, including Program to Encourage Active, Rewarding Lives for Seniors (PEARLS) [43] or Improving Mood-Promoting Access to Collaborative Treatment (IMPACT) [44], both involving education, counseling, and social activities. However, both programs are limited because they do not consider the differences in physical and mental characteristics between the young-old and the old-old subgroup. It would be more helpful for future programs if they account for the impacts of different social support types depending on the age group. The difference in the importance of family support across different age groups may imply that practitioners should help couples communicate better regarding the caregiver roles in late life. Regular contact with intimate family members, in addition to spouse support for young-old adults, may also improve or prevent depression, which could be a practice encouraged by practitioners.

There were also results that contradicted previous findings. Guo et al. [45] reported that spousal support did not predict mental health outcomes, and that family supports were actually harmful for U.S. Chinese older adults’ well-being. Their mixed findings may be explained by the shift in the immigration process. Family interdependence may potentially harm older immigrant adults because it may undermine their sense of self, and immigrant family dynamics may have been changed from the traditional family structure.

This study has some limitations. First, the findings may not apply to the older populations who have different cultural backgrounds. For instance, older Asian immigrants may prefer to live with or close to their adult children, and family support might play a more prominent role than spousal support for this group. In Asian culture, the virtue of filial piety asks for adult children to fulfill the needs of their older parents, and works as a protective factor against depression in this group [46,47]. Second, the research only represents Medicare beneficiaries aged 65 or older, those without severe cognitive impairment, and those able and willing to complete the survey. Follow-up research should incorporate responses for older adults with cognitive disabilities and arrange ways to ensure their full participation.

Future researchers may explore whether the findings from this study are replicable in other contexts, such as with larger samples, higher response rates, and in-depth interviews. Because non-white races comprised only a small percentage in this study (Asian: 3.7%; Black: 16.4%; Hispanic: 11.4%), it is not clear that the findings are generalizable to other ethnic and cultural groups. Moderating effects from different sources of social support should be further examined. Because of the limitations in measuring comprehensive social support using secondary data, the measures of social support were limited to spouse and family support. Other types of social support, such as from significant others, neighbors, or community members, should also be considered when measuring social support [22,48].

## 5. Conclusions

Social support is a crucial factor in preventing loneliness and depression for older adults, and spousal support plays a positive role in seniors’ mental health. As the role of caregivers in a person’s well-being grows later in life, it would be beneficial for practitioners to encourage couples to communicate with this consideration. Furthermore, regular contact with family members and spousal support would be helpful to prevent older old adults’ depression.

## Figures and Tables

**Table 1:** Descriptive Statistics of the Young-Old and the Old-Old.

Variables		The Young-Old (*n* = 903)		The Old-Old (*n* = 629)	
		*n*	%	*n*	%
Gender	Male	463	51.3	376	59.8
	Female	440	48.7	253	40.2
Age	M (SD)	69.79 (2.58)		80.21 (4.17)	
Education Attainment	Less than High School	106	11.7	96	15.3
	High School Equivalent	193	21.4	157	25.0
	Vocational Certificate/Some Degree/Associates	307	34.0	196	31.2
	Bachelors or More	297	32.9	180	28.6
Race	White	708	78.4	480	76.3
	Other	195	21.6	149	23.7

**Table 2:** Differences in Main Variables between the Young-Old and the Old-Old.

Variables	The Young-Old (n = 903)		The Old-Old (n = 629)		t
	M	SD	M	SD	
Loneliness	0.86	0.73	0.87	0.67	−0.126
Spouse Support	2.29	0.50	2.26	0.51	1.448
Family Support	2.19	0.52	2.23	0.52	−1.253
Depression	1.41	0.42	1.45	0.42	−1.800

**Table 3:** Multivariate Regression Models of Depression among Participants.

Variables		The Young-Old (*n* = 903)		The Old-Old (*n* = 629)	
		B	S.E.	B	S.E.
Constant		1.608	0.334	0.510	0.306
Control Variable	Gender (ref. male)	0.087 **	0.025	0.089 **	0.032
	Age	−0.001	0.005	0.012 **	0.004
	Education Attainment	−0.055 ***	0.013	−0.024	0.015
	Race (ref. white)	−0.072 *	0.031	−0.037	0.037
Independent Variable	Loneliness(A)	0.195 ***	0.018	0.161 ***	0.026
Moderator Variable	Spouse Support(B)	−0.127 ***	0.029	−0.156 ***	0.032
	Family Support(C)	−0.060 *	0.027	0.015	0.033
Interaction	A × B	−0.050	0.036	−0.092 *	0.042
	A × C	−0.043	0.032	0.003	0.041
R^2^		0.249		0.187	
F(sig.)		32.941 ***		15.816 ***	

* *p* < 0.05, ** *p* < 0.01, *** *p* < *0*.001.

## Data Availability

Not applicable.
